# Advancements in Anterior Cruciate Ligament Injury: From Diagnosis to the Return-to-Sports

**DOI:** 10.3390/jcm14030722

**Published:** 2025-01-23

**Authors:** Nicolas Bouguennec, Alexandre Hardy

**Affiliations:** 1 Clinique du Sport de Bordeaux-Mérignac, 33700 Mérignac, France; 2 Clinique du Sport, Universidad de París V Descartes, 75005 Paris, France; alexandre.hardy@me.com

The anterior cruciate ligament (ACL) plays a crucial role in knee stability and function, with its injuries posing challenges across the domains of prevention, treatment, and rehabilitation. This Special Issue, “Advancements in Anterior Cruciate Ligament Injury,” features a selection of five impactful studies that highlight the current trajectory of research and clinical practice in ACL management. These contributions, chosen for their relevance and innovative approaches, underscore the importance of a multidisciplinary framework in addressing the complexities of ACL injuries.

Prevention remains a cornerstone of ACL injury management, particularly in high-risk sports involving jumping, pivoting, and rapid directional changes. Research included in this Special Issue, particularly the systematic review by Antoranz et al. (Contribution 1), examines the effectiveness of targeted injury prevention strategies among basketball players, a population frequently exposed to ACL injuries. By focusing on biomechanical training and neuromuscular conditioning, this work highlights the potential of tailored interventions to reduce injury rates, enhance athletic performance, and mitigate the long-term complications associated with ACL damage.

Knee alignment, while less commonly discussed, is a highly influential factor in ACL injuries and their surgical outcomes. This Special Issue includes research by Onishi et al. (Contribution 2) on the use of infratubercle anterior closing wedge osteotomy to correct sagittal malalignment without compromising coronal alignment or patellar height. The study’s focus on achieving biomechanical harmony through innovative surgical techniques aligns with the broader goal of restoring knee function while minimizing post-operative complications.

Additionally, graft selection and preparation—critical components of ACL reconstruction—are explored through a comparative study of Achilles tendon allograft preparation techniques. This research, led by Thiel et al. (Contribution 3), emphasizes tensile strength optimization to meet the mechanical demands placed on grafts, particularly in active populations. By providing insights into preparation methods, this study directly contributes to the refinement of surgical practices and the improvement of patient outcomes.

The surgical management of ACL injuries has also evolved significantly, with revision ACL reconstruction presenting unique challenges due to a compromised biomechanical environment. The study by Jung et al. (Contribution 4) investigates the impact of combined anterolateral ligament (ALL) and ACL reconstruction techniques, offering evidence that this approach enhances rotational stability and patient-reported outcomes. By addressing one of the most persistent issues in revision surgery, i.e., graft failure, this research broadens the scope of surgical innovation and emphasizes patient-centered care. The authors conclude that combining ALL reconstruction with revision ACL reconstruction in non-athletes results in a higher proportion of patients experiencing less mechanical graft failure and achieving satisfactory clinical outcomes.

Finally, rehabilitation and return-to-sport (RTS) protocols are critical components of the ACL recovery journey. A compelling study by Gerfroy et al. (Contribution 5) investigates the predictive value of RTS assessments conducted six months post-surgery on long-term functional outcomes. These findings highlight the importance of early milestones in shaping recovery trajectories and reinforce the need for precise and individualized rehabilitation strategies to optimize mid- and long-term outcomes.

This Special Issue was designed to reflect the multifaceted nature of ACL injury management, encompassing prevention, surgical advancements, rehabilitation, and the optimization of associated factors such as alignment and graft mechanics. The selected articles provide a cohesive narrative that highlights the interplay between innovative research and clinical application, reinforcing the importance of an integrated approach to addressing ACL injuries.

As the field progresses, it is imperative to continue fostering collaborations among researchers, clinicians, and rehabilitation specialists to ensure that advancements are translated into tangible improvements in patient care. By addressing key challenges and proposing forward-thinking solutions, the studies featured in this Special Issue contribute meaningfully to the ongoing evolution of ACL management, offering hope for improved outcomes and quality of life for those affected by these injuries.

